# The Synergistic Effect of Time of Exposure, Distance and No Use of Personal Protective Equipment in the Determination of SARS-CoV-2 Infection: Results of a Contact Tracing Follow-Up Study in Healthcare Workers

**DOI:** 10.3390/ijerph18189456

**Published:** 2021-09-08

**Authors:** Giuseppe La Torre, Mattia Marte, Carlo Maria Previte, Lavinia Camilla Barone, Filippo Picchioni, Marta Chiappetta, Augusto Faticoni, Daniela Marotta, Elena Mazzalai, Vanessa India Barletta, Shizuka Kibi, Vittoria Cammalleri, Barbara Dorelli, Monica Giffi, Roberta Noemi Pocino, Anna Paola Massetti, Caterina Fimiani, Ombretta Turriziani, Ferdinando Romano, Guido Antonelli, Alberto Deales, Claudio Maria Mastroianni, Fortunata Vasaturo, on behalf of Collaborative Group Sapienza

**Affiliations:** 1 Department of Public Health and Infectious Diseases, Sapienza University of Rome, 00185 Roma, Italy; mattia.marte@uniroma1.it (M.M.); carlomaria.previte@uniroma1.it (C.M.P.); lavinia.barone@uniroma1.it (L.C.B.); picchioni.1697595@studenti.uniroma1.it (F.P.); marta.chiappetta@uniroma1.it (M.C.); augusto.faticoni@uniroma1.it (A.F.); daniela.marotta@uniroma1.it (D.M.); elena.mazzalai@uniroma1.it (E.M.); barletta.1325951@studenti.uniroma1.it (V.I.B.); kibi.1041042@studenti.uniroma1.it (S.K.); vittoria.cammalleri@uniroma1.it (V.C.); barbara.dorelli@uniroma1.it (B.D.); monica.giffi@uniroma1.it (M.G.); robertanoemi.pocino@uniroma1.it (R.N.P.); paola.massetti@uniroma1.it (A.P.M.); catfim@yahoo.com (C.F.); ferdinando.romano@uniroma1.it (F.R.); claudio.mastroianni@uniroma1.it (C.M.M.); 2 Unit of Occupational Medicine, Teaching Hospital Policlinico Umberto I, 00161 Roma, Italy; 3 Laboratory of Virology, Department of Molecular Medicine, Sapienza University of Rome, 00185 Roma, Italy; ombretta.turriziani@uniroma1.it (O.T.); guido.antonelli@uniroma1.it (G.A.); 4 Health Direction, Teaching Hospital Policlinico Umberto I, 00161 Roma, Italy; a.deales@policlinicoumberto1.it; 5 Clinica Medica Department of Internal Medicine and Medical Specialties, Sapienza University of Rome, 00185 Roma, Italy; f.vasaturo@policlinicoumberto1.it

**Keywords:** SARS-CoV-2, COVID-19, contact tracing, safety, synergism, personal protective equipment, distance, time of exposure

## Abstract

The aim of this study is to assess the effect of contact time, contact distance and the use of personal protective equipment on the determination of SARS-CoV-2 infection in healthcare workers (HCWs). This study consists of an analysis of data gathered for safety reasons at the Sapienza Teaching Hospital Policlinico Umberto I in Rome through the surveillance system that was put into place after the worsening of the COVID-19 pandemic. The studied subjects consist of HCWs who were put under health surveillance, i.e., all employees who were in contact with subjects who were confirmed to have tested positive for SARS-CoV-2. The HCWs under surveillance were monitored for a period encompassing ten days after the date of contact, during which they undertook nasopharyngeal swab tests analysed through RT-PCR (RealStar® SARS-CoV-2 Altona Diagnostic–Germany). Descriptive and univariate analyses have been undertaken, considering the following as risk factors: (a) no personal protective equipment use (PPE); (b) Distance < 1 m between the positive and contact persons; (c) contact time > 15′. Finally, a Cox regression and an analysis of the level of synergism between factors, as specified by Rothman, were carried out. We analysed data from 1273 HCWs. Of these HCWs, 799 (62.8%) were females, with a sample average age of 47.8 years. Thirty-nine (3.1%) tested positive during surveillance. The overall incidence rate was 0.4 per 100 person-days. Time elapsed from the last exposure and a positive RT-PCR result ranged from 2 to 17 days (mean = 7, median = 6 days). In the univariate analysis, a distance <1 m and a contact time > 15′ proved to be risk factors for the SARS-CoV-2 infection, with a hazard ratio (HR) of 2.62 (95% CI: 1.11–6.19) and 3.59 (95% IC: 1.57–8.21), respectively. The synergism analysis found the highest synergism between the “no PPE use” x “Contact time”. The synergy index S remains strongly positive also in the analysis of the factors “no PPE use” x “Distance” and “Time of contact” x “Distance”. This study confirms the absolute need to implement safety protocols during the pandemic and to use the correct PPE within health facilities in order to prevent SARS-CoV-2 infection. The analysis shows that among the factors considered (contact time and distance, no use of PPE), there is a strong synergistic effect.

## 1. Introduction

Prompt identification, isolation and contact tracing are common interventions to control infectious disease outbreaks; they can be effective but might require intensive public health efforts and cooperation to reach and monitor all contacts for safety reasons [1]. In the fight against the SARS-CoV-2 outbreak, these tools have become especially important because of the presence of asymptomatic and paucisymptomatic subjects amongst those affected by the virus. More specifically, contact tracing has allowed Healthcare workers (HCWs) to identify potentially infected individuals before the onset of symptoms, and, in the case of early identification, prevent subsequent transmission to secondary cases [1]. The Federal State of Germany’s decision on the 6th of May, 2020, to reopen society in a stepwise fashion was taken considering its expanded capacity for contact tracing, exemplifying how crucial this tool has been to counter the spread of SARS-CoV-2 [2].

In Italy, contact tracing is performed in two ways, either through an epidemiological investigation or through a computerized system. The former consists of an interview of the positive subject where he/she is asked about any contacts in the last 72 h, as per the incubation period of 4 to 6 days [3]. The latter consists of the use of the Immuni App, which, thanks to Bluetooth geolocation, allows HCWs to identify those who have had probable contact with a subject who later became positive [4]. Both methods are regulated by the Prime Minister’s Decree of the 14th of January 2021. Article 5 [5], specifically, notes that in the presence of a positive subject, the healthcare worker of the Local Health Authority is legally obliged to upload the subject’s key code in the Immuni App. More generally, in the same Decree, article 8, paragraph 5, it is reported that the healthcare worker and the territorially competent Public Health Services will prescribe quarantine for a subject through the following procedure:

(A) Contacting the subject by phone and asking for the relevant information and documents regarding their area of residence and any travel done in the previous 14 days to evaluate their risk exposure; (D) ascertaining the absence of fever or other symptoms in the subject as well as in any other cohabitants; (E) informing the person about the symptoms, the characteristics of contagiousness, the mode of transmission of the disease, and the measures to be implemented to protect any cohabitants in case of appearance of symptoms; (F) informing the person about the need to measure their body temperature twice a day (in the morning and in the evening), as well as to maintain the state of isolation for fourteen days from the last exposure, the prohibition of social contacts, the ban on travel, and the obligation to remain reachable for surveillance activities; (H) contacting the person under surveillance daily for information on health conditions. In the event symptoms appear, the public health doctor proceeds in accordance with the provisions of circular no. 5443 of the Ministry of Health of the 22nd of February 2020. This is conducted only after having consulted the subject’s general practitioner or their paediatrician [5].

From an occupational point of view, contact tracing is also an important source of data for the analysis of the spread and manifestation of the virus amongst HCWs and its determinants. In the healthcare sector, contact tracing can be considered one of the most important safety issues, especially before the availability of COVID-19 vaccines. Therefore, the aim of this study is to assess the synergistic effect that the time of exposure, distance from the SARS-CoV-2 positive, and use of personal protective equipment have on the spread and manifestation of the SARS-CoV-2 infection amongst HCWs. These determinants have been taken from the WHO’s “Advice for the public”, where important factors relating to the spread of the virus amongst the population were identified: environment, distancing, and time of exposure [6].

## 2. Materials and Methods

Between March 2020 and November 2020, following the worsening of the COVID-19 epidemic, the Policlinico Umberto I Teaching Hospital put a surveillance system into place to prevent the spread of the virus amongst HCWs. All employees, including post-graduate doctors, students, interns, trainees, cleaners, and canteen staff, were put under surveillance. 

The presence of SARS-CoV-2 in the subjects was tested by performing nasopharyngeal swabs at the Hospital Outpatient Infectious Disease Service. Viral transport media (VTM) was used by trained physicians and nurses to transfer each specimen into a 3 mL vial. Specimens were either transported immediately or refrigerated at 2–8 C. They were also refrigerated during transport. Testing was carried out at the Laboratory of Virology in the hospital, which is located no more than 100 m from the Outpatient Infectious Disease Service. After RNA extraction, diagnosis of SARS-CoV-2 infection was performed by RT-PCR (RealStar® SARS-CoV-2 Altona Diagnostic–Germany). This procedure for nasopharyngeal swabs was performed with an automated sample preparation (SP) module using a Versant SP 1.0 Reagents kit (Siemens Healthcare Diagnostics Inc., Tarrytown, NY, USA). The limit of detection was 1000 cp/mL.

Following the notification of a newly positive subject, the heads of the hospital’s departments were asked to conduct an epidemiological investigation for which they had to fill out specific contact forms (attachment 1) detailing the names of all the HCWs who had contact with the positive subject in the 72 h prior to the insurgence of symptoms/a positive nasopharyngeal swab test. The forms included other important information such as: personal data, mobile number, email address, hospital unit, organization, professional role, date of contact with the positive subject, usage of personal protective equipment (Yes/No), distance from the positive subject (more than/less than 1 m) and time of exposure (more than/less than 15 min). Subjects put under surveillance were monitored for 10 days to allow for the identification of any potential insurgence of the infection. The protocol that was put into place mandated for at least one nasopharyngeal swab test to be taken. More specifically, swab tests were to be conducted at T0 (the day in which/the day after the subject met) at T1 (five days after T0) and at T2 (ten days after T0). Whenever the notification of contact was sent between three or seven days after the date of contact, two tests were taken. Only one swab was necessary if notification was made after the eighth day.

A descriptive analysis of the sample was performed. The univariate analysis involved the use of the chi-square test and the Student’s t-test, respectively, to evaluate the differences for qualitative and quantitative variables. Furthermore, a Cox regression analysis was performed to evaluate the association/correlation between the variables: distance from the positive subject, protective equipment use (PPE) use, and time of exposure. The analysis was adjusted for gender, age and professional role, and the results were expressed through a Hazard Ratio (HR) and 95% CI. Finally, the synergistic effect between the variables was evaluated through an analysis that was conducted according to the methodology described by Rothman [7]. The “synergy index” was calculated as follows (Equation (1)):

S = [HR_11_ − 1]/([HR_01_ + HR_10_] − 2), (1)

where HR_11_ is equal to the HR of the joint effect of two risk factors, and HR_10_ and HR_01_ are equal to the HR of each risk factor in the absence of the other. Additivity was considered for the value of S equal to 1, whereas superadditivity and synergism were present for values of S over 1 [7]. The statistical analysis software used is SPSS v 25.0 (IBM, Armonk, NY, USA). The significance level has been set to *p* < 0.05. 

## 3. Results

We analysed data from 1273 HCWs undergoing health surveillance because a worker tested positive for the nasopharyngeal SARS-CoV-2 swab test. Of these operators, 799 (62.8%) were female, and 474 (37.2%) were male, with a sample average age of 47.8 years. During surveillance, we found 39 HCWs positives (3.1%; 23 female and 16 male), of which 24 (61.5%) were identified after the first swab and 15 (38.5%) were identified after the second swab.

The overall incidence rate during our health surveillance activity was 0.4 per 100 person-days. Time elapsed from the last exposure, and the positive RT-PCR result ranged from 2 to 17 days (mean = 7, median = 6 days). In Table 1, we described demographic and professional characteristics of the working population, and we performed a univariate analysis for the variables use of the PPE, distance and contact time. When looking at the professional role of those who tested positive, 20 were nurses (4.5%), 9 doctors (1.8%), 10 other health workers (3.2%), and no administrative workers (*p* = 0.08). Concerning the variables analyzed, the rate of positives was double (5.6%) in HCWs who did not use PPE compared to those who used them (2.8%), triple (4.4)% in workers who had contact at a distance less than 1m compared contacts at more than 1m (1.4%). Moreover, considering the variable contact time, we obtained a rate of 5.3% for contact that lasted more than 15 min and a rate of 1.4% for those that lasted less than 15 min (*p* < 0.001).

In Table 2, we show the results of the Cox regression analysis, adjusted for gender, age, and job role. It shows that for a contact time greater than 15 min, the HR = 3.59 (95% IC: 1.57–8.21), and for a distance less than 1 m, the HR = 2.62 (95% IC: 1.111–6.19), and for the non-use of PPE, the HR = 1.62 (95% IC: 0.67–3.92). Regarding the analysis of synergism, the index shows a synergistic effect for all combinations of risk factors, with a higher index for the combination of PPE and contact time (S = 5.2), followed by the combination between the use of PPE and contact distance (S = 2.82) and the combination between contact distance and contact time (S = 1.29).

## 4. Discussion

The incidence of positives among HCWs in our experience is similar to that found by Lahner et al. [8] and much lower if compared to what was found by Scozzari et al. and Fong et al. [9,10], testifying the effectiveness of the preventive measures. The percentage of positives among supervised operators is double among nurses compared to doctors (respectively, 4.5% vs. 1.9%), a trend also found in a Chinese study concerning the first months of the pandemic [11]. This is probably due to the greater time of contact those nurses have with patients, although this difference is statistically insignificant.

Our results relating to the difference between the positivity date and the date of the last contact (7 days) are in accordance with what is reported by Coppeta et al., which identify an average of 6.7 days [1]. The lack of infected subjects among the supervised workers in the administration highlights how health care increases the risk of contagion (0 vs. 39). Of the positives (39), those who were at less than 1 m from the positive subject were significantly found to be four times the number of those at a distance greater than 1 m. As for the contact time, 29 of the operators who tested positive had an exposure greater than 15 min, compared to 10 positive results in contact for less than 15 min. Predictably, those who had contact with a patient or HCWs without wearing PPE doubled the likelihood of being positive, compared to those who wore PPE (5.6% vs. 2.8%). Therefore, we agree with Chung et al., whose results show that the use of PPE protects against contagion, guaranteeing a lower risk of infection [12]. Gender does not affect the risk of contagion. Even if there is evidence that female HCWs are at higher risk of contracting the disease [13], our results are overlapping with those coming from the Italian general population [14].

Luca Coppeta et al. did not find statistical significance in maintaining a distance greater than 2 m [1]. In contrast, Seidelman et al. state that the absence of the surgical mask induces a 70% greater risk of contagion when combined with a duration of exposure greater than 10 min and less than 1.829 m (6 feet) [15]. Although with different values, Seidelman et al. are therefore in agreement with our analysis. Concerning the proximity in the transmission model of the SARS-CoV-2, the choice of one meter as a reference measure for assessing the exposure is evidence-based. As suggested by Buxton et al., a decrease of the distance from 2 m to 1.25 m is associated with an increase of the exposure to the viral particles by a factor of 26 [16]. Moreover, according to Bu et al., airborne transmission is a significant route in both short-and long-range virus transmission. Physical distancing recommendations reported in this review go from 1 m (infection risk under 0.63) to 3 m (Infection probability under 2%) [17]. In relation to the contact time, our results agree with what was found by Chen et al., who established that an exposure greater than 30 min and a distance less than 1 m induces an increased risk of contagion [18]. The synergy analysis shows a very high value if we take into consideration the relationship between the factors “PPE use” and “Contact time”, with a value of S equal to 5.20. This data is particularly important if we consider that in the univariate analysis, the relationship between the non-use of PPE and positivity was not significant. The combined effect of non-use of PPE and contact time greater than 15 min, therefore, confirms the importance of the use of PPE in a hospital setting where the contact time in assistance activities is likely to exceed 15 min. The importance of PPE usage in the protection of HCWs who cannot maintain a distance of at least 1 m to patients is further supported by the synergism that we have identified between the factors “Use of PPE” and “Distance”, with a value of S equal to 2.82. The synergy value between the two factors that do not include PPE (distance and time) has a value of S = 1.29, implying a synergistic effect that is certainly present but not of the same magnitude that we find in the analyses that include the “PPE use” factor.

### Limitations

However, this paper has some limitations. First, the use of PPE was considered as aggregate data, i.e., the differences between the type of mask (surgical or FFP2), the use of gloves, protective goggles, headphones, gowns were not detected. Similarly, the differences between work settings (clinics, wards, intensive care), which could influence the risk of contagion, were not defined in the study. A further element to consider is undoubtedly the misclassification of exposure: it is the contact person (nursing coordinator or ward manager) of the department involved who compiles the operator contact form based on the data provided both by the index case and by the health professionals who came into contact with the same.

## 5. Conclusions

Overall, we can conclude from a safety point of view that this study confirms the absolute need to use the correct PPE in the health sector for the prevention of SARS-CoV-2 transmission. Furthermore, the analysis shows that among the factors considered (contact time and distance, use of PPE), there is an evident synergistic effect, particularly marked if we consider the combination of time factors and use of PPE.

## Figures and Tables

**Table 1:** The results of the univariate analysis. N = Number of HCWs; SD = Standard deviation.

	Positive N (%) or Mean (SD)	Negative N (%) or Mean (SD)	*p*
Gender			
F	23 (2.9)	776 (97.1)	0.619
M	16 (3.4)	458 (96.6)	
Age (years)	47.8 (16.5)	48.1 (17.3)	0.915
Role			
Doctor	9 (1.9)	457 (98.1)	
Nurse	20 (4.5)	424 (95.5)	0.080
Other medical staff	10 (3.2)	302 (96.8)	
Administrative	0 (0)	51 (100)	
Use of the protective equipment			
No	8 (5.6)	136 (94.4)	0.072
Yes	31 (2.8)	1081 (97.2)	
Distance			
More than 1 metre	8 (1.4)	546 (98.6)	0.003
Less than 1 metre	31 (4.4)	672 (95.6)	
Contact time			
Less than 15 min.	10 (1.4)	703 (98.6)	<0.001
More than 15 min.	29 (5.3)	515 (94.7)	

**Table 2:** Hazard ratio values by the combination of risk factor and synergy index.

Variables	HR	Synergism
Distance x time		
Distance < 1 m—time > 15 min	13.11 (3.04–56.6)	
Distance < 1 m—time < 15 min	5.37 (1.14–25.3)	1.29
Distance > 1 m—time < 15 min	6.04 (1.17–31.2)	
Distance x use of PPE		
Distance < 1 m—Use of device No	13.5 (4.08–44.7)	
Distance < 1 m—Use of device Yes	3.77 (1.42–10.07)	2.82
Distance > 1 m—Use of device No	2.66 (0.51–13.81)	
Use of PPE x time		
Use of device No—time < 15 min	5.73 (2.24–14.69)	
Use of device No—time > 15 min	2.91 (1.31–6.49)	5.20
Use of device Yes—time > 15 min	0	

## Data Availability

Data will be available upon request to the correspondence author due to privacy/ethical restrictions.
